# Why are we awake? Algorithmic serendipity and the sociology of sleeplessness

**DOI:** 10.3389/fsoc.2025.1492373

**Published:** 2025-09-05

**Authors:** Boroka Bo, Aidan O’Sullivan, Eva Bariol, Nicole Kucherenko, Molly Behan

**Affiliations:** ^1^Sociology, University College Dublin, Dublin, Ireland; ^2^Sociology, University of California Berkeley, Berkley, CA, United States

**Keywords:** insomnia, algorithmic serendipity, community building, YouTube, sleeplessness, human-technology interactions

## Abstract

Algorithmic serendipity is the seemingly chance encounter with exactly the right content, engineered by online recommender systems, linking individuals to one another and to digital remedies. This phenomenon transforms the individual experience of insomnia into a collective experience, creating communities around shared sleeplessness. Using a corpus of YouTube comments, we present a comprehensive theory of how algorithmic mediation may reshape insomnia in late modernity. Insomniacs forgo rest to read and share their experiences with sleeplessness, forging community instead of sleep. In tracing this loop, we show how disclosure practices, peer validation, and platform logic fuse to turn a private symptom into a shared social condition. The result is a paradox: the same digital infrastructures that soothe wakeful nights sustain insomnia. Recognizing this underscores that digital culture must sit at the center of sleep research. Interventions should target not only individuals, but the collective rhythms and norms that animate digital worlds.

## Introduction

This study explores how ‘algorithmic serendipity’ shapes the collective routines of individuals struggling with insomnia and, in turn, reshapes their nights. Algorithmic serendipity refers to when a recommendation system, cued by what users do, serves up content that feels like pure happenstance yet is the product of shared human–machine labor; say, a stream of sleep music at 2 a.m. (Zhang et al., 2012; André et al., 2009; Beale, 2007). Insomnia is chronic difficulty initiating, maintaining, or timing sleep, leading to next-day impairment. As a transient symptom it appears whenever stressors, medications, or illness disrupt rest. As a disorder it occurs at least three nights a week for 3 months or more (Harvey, 2001). Yet, insomnia is more than just an individual phenomenon. A growing body of literature shows that sleeplessness is deeply social: society sustains sleep, while at the same time, sleep sustains society (Kim et al., 2021; Crary, 2013; Williams, 2008). Yet, this bargain is fraying: since the early twentieth century, average nightly sleep has shrunk by nearly 4 h (Meadows, 2019; Walker, 2017; Crary, 2013; Williams, 2002).

Insomnia exacts private and public costs, reverberating through work, family, and civic life (Araújo et al., 2017). Sleeplessness increases fatigue, irritability, anxiety, depression, and disability rates (Hillman and Lack, 2013; Léger and Bayon, 2010; Daley et al., 2009; Buysse et al., 2006). These repercussions shape how individuals interact with their communities, with insomniacs reporting lower rates of community participation and belonging (Kim et al., 2021; Robbins et al., 2019). Despite insomnia’s physical, mental, and social toll, most individuals prefer to self-manage their condition via bedtime rituals that often involve streaming media such as YouTube (Dickson and Schubert, 2019; Léger and Bayon, 2010; Ohayon, 2002).

Knowing the above, programmers build recommendation engines that usher users toward tailored sleep tracks, amplifying algorithmic serendipity—the seemingly fortuitous discovery of targeted streaming media forged by code and clickstream (Zhang et al., 2012; André et al., 2009; Beale, 2007). The term foregrounds how agency and chance intertwine as people manage insomnia. Yet scholars still lack a coherent account of how sleeplessness is produced and sustained, even after noting that digital technologies blur public–private and individual–collective life (Crary, 2013; Williams, 2011; Schor, 2008; Henry et al., 2008). The stakes are high. Our experience of modernity is global, yet fragmented (Matin, 2021): we coexist ‘within modernized spaces and speeds, yet simultaneously inhabit the remnants of pre-capitalist life-worlds, whether social or natural’ (Crary, 2013, p. 66). The social and contextual causes and consequences of sleeplessness are under-examined, especially in settings where the social organization of modernity and associated norms of productivity and labor may differ (Williams, 2011).

Integrating prevalent theoretical perspectives from the biopsychology and sociology of sleep, research from science and technology studies, and from literature on online community participation allows us to illuminate the sociological paradox of our collective insomnia in the digital age. We show that insomnia creates the conditions for two specific forms of algorithmic serendipity. Algorithmic serendipity then shapes how individuals struggling to sleep discover and create online co-sleeping communities. Their individual and community-based needs influence associated norms of anonymous online information disclosure. As sleep-deprived music listeners unexpectedly find themselves both reading about other’s experiences and disclosing their own experiences with the disorders undergirding insomnia, this prevents them from falling asleep. Lacking supportive offline communities, instead of sleeping, they engage in community participation and the co-construction of belonging. Paradoxically, their successful efforts to ameliorate the original factors underpinning their insomnia animate their insomnia.

## Sleepless in modernity

A growing body of literature on the individual and social experience of sleep highlights that our sleep mirrors the spatially disjointed and temporally arrhythmic experience of late modernity (Meadows, 2019; Crary, 2013; Williams, 2002). Our sleep time is dwindling: our parents’ generation slept for 8 hours, down from 10 h in the beginning of the 20th century. Globally, about seven to 10 % of adults meet the medical diagnostic criteria for insomnia disorder, while one in four individuals report experiencing episodic insomnia (Van Someren, 2021; Soldatos et al., 2005; Roth and Roehrs, 2003). Residents of North America are currently sleeping a little over 6 h per night (Walker, 2017; Crary, 2013). In the United States, approximately 60 percent of adults report episodic sleeplessness, experiencing trouble falling or staying asleep multiple times per week (Anon, 2024b).

These alarming statistics reflect deeper structural transformations in how sleep functions within our society. As modernity intertwined labor and time, the experience of sleep also morphed: from rest to the rehabilitation of ‘a coherent and functional (capitalist) self’ (Meadows, 2019, p. 259). In support of this, researchers have shown that focusing on individual behavior is inadequate for explaining the prevalence of insomnia. Sleeplessness is driven by our inability to stop producing and consuming, with work spilling into our private lives via cognitive labor (Henry et al., 2008; Williams, 2011; Crary, 2013; Giddens, 2020). High work-related stress, unhealthy organizational environments, and low coworker support contribute to insomnia (Nakata et al., 2004). As such, sleeplessness becomes a 24-h problem, disturbing all domains of life (Araújo et al., 2017).

Relatedly, social scientists note that sleeplessness needs to be examined in the context of late modernity blurring distinctions between public-private and individual-collective spheres (Crary, 2013; Williams, 2011; Schor, 2008; Henry et al., 2008). Insomnia resides in the liminal space between public and private: we lay awake, vigilant and ruminating, yet the individuals and events occupying our thoughts remain outside our homes (Levinas, 1978). Blurring individual and collective, public health professionals inform us that we should be preoccupied with our ‘sleep hygiene,’ urging us to refrain from stimulating activities before bedtime (Coveney et al., 2023; Espie, 2022; Wolf-Meyer, 2008; Williams and Boden, 2004). Not surprisingly, empirical research shows that sleep-conducive physical environments are beneficial for reducing insomnia (Nakata et al., 2004). Similarly, unwanted noises can cause sleep disturbances (Ong et al., 2014). In sum, the individual experience of insomnia is concurrently deeply social: sleep stands ‘for the durability of the social… analogous to other thresholds at which society could defend or protect itself. As the most private, most vulnerable state common to all, sleep is crucially dependent on society in order to be sustained’ (Crary, 2013, p. 25).

So far, we have shown that sleep—or the lack of it—is dependent on society. Society, however, is also dependent on sleep. Sleeplessness comes with both direct and indirect social costs. For example, insomnia leads to fatigue, irritability, and an inability to perform simple tasks (Hillman and Lack, 2013; Daley et al., 2009; Buysse et al., 2006; Roth and Roehrs, 2003). Insomnia is likewise associated with anxiety, depression, and disability (Léger and Bayon, 2010). These repercussions shape how people interact with their communities (Kim et al., 2021). Individuals who sleep less than 7 h per night are more likely to report decreased community participation and belonging (Robbins et al., 2019). Sleeplessness also increases work absenteeism while decreasing public safety (Rosekind and Gregory, 2010; Léger et al., 2006), resulting in an estimated $35 billion annual cost to US employers (Chilcott and Shapiro, 1996).

## Agency and serendipity in sleeplessness

The above literature on how insomnia influences behavioral outcomes alerts us to the necessity to consider individual agency in relation to sleeplessness. Sociologists note that individual agency plays a significant role in regulating sleep patterns and facilitating both the transition into sleep and the awakening process (Taylor, 1993). For instance, ‘pre- and post-sleep routines’ serve as ‘transitional practices and symbolic markers,’ used to navigate the daily transition between our conscious and unconscious states (Williams, 2008, p. 642). In other words, bedtime habits under an individual’s control can trigger or worsen insomnia (Stepanski and Wyatt, 2003).

Reflecting a preference for agency, individuals tend to favor managing their insomnia without the aid of medical professionals. However, prevalence estimates for sleeplessness vary dramatically depending on how insomnia is defined and measured. Clinical diagnostic criteria for insomnia disorder, which require specific frequency, duration, and impairment thresholds, indicate global prevalence rates of seven to 10 % (Van Someren, 2021; Roth and Roehrs, 2003). In contrast, broader definitions that include any self-reported sleep difficulties or episodic insomnia produce much higher estimates, ranging from 25 percent globally to 60 percent in the United States (Anon, 2024b). These estimates reflect not only methodological differences but also cultural variations in how sleep problems are conceptualized and reported.

This definitional complexity may partially explain why so few people with severe insomnia ever visit a doctor for this complaint. Léger and Bayon (2010) show that medical consultation rates range from 5% to approximately half, depending on cultural context. Instead, many choose to consume streaming content, watching television or online videos until they fall asleep (Dickson and Schubert, 2019; Ohayon, 2002). Although researchers are still grappling with how streaming media influences sleeplessness, recent work shows that listening to relaxing music can improve sleep quality (Eke et al., 2020; Cordi et al., 2019).

This turn toward digital self-management has created new opportunities for technological intervention in sleep practices. Noting the importance of both agency and chance in how people find online information, computer scientists have been grappling with designing algorithms to boost the serendipitous encounter of specific online content, such as sleep music (Zhang et al., 2012; Beale, 2007). Algorithmic serendipity refers to the phenomenon where individuals encounter unexpected but relevant content through the workings of an algorithm. Unlike traditional serendipity, which happens by chance (Lieblich et al., 2008), this occurs as algorithms curate content based on user behavior, preferences, and interaction history (André et al., 2009).

For the large percentage of insomniacs self-managing their disorder, this algorithmic curation becomes particularly significant (Anon, 2024b; Dickson and Schubert, 2019; Léger and Bayon, 2010; Ohayon, 2002). The consideration of algorithmic serendipity underscores how agency and chance intertwine as individuals manage their insomnia (Dickson and Schubert, 2019). This is not a passive process: users actively engage with streaming content, providing feedback through their interactions, which refines the algorithm’s future recommendations (André et al., 2009). As we will shortly show, the dynamic interaction between individual agency and algorithmic serendipity influences how insomniacs understand and experience their sleeplessness, in addition to shaping how they construct and engage with their communities.

## (not) sleeping together

What is the relationship between sleeplessness, agency as it is shaped by algorithmic serendipity, and community engagement? This question is important, as it highlights a tension in the literature: While sleeplessness decreases offline community engagement (Kim et al., 2021; Robbins et al., 2019; Kyle et al., 2010), we also know that algorithmic serendipity may aid engagement with online communities (Rotman et al., 2009; Koh et al., 2007). Similarly to offline communities, online communities can also be highly cohesive. They permit the formation and exchange of distinct types of social capital, composed of ‘networks, norms and trust’ enabling ‘participants to act together more effectively to pursue shared objectives’ (Putnam, 1995, p. 67). They likewise bolster well-being by providing emotional, practical, and cognitive support (Miller, 2020; Rotman et al., 2009). Online community members share information, develop lasting relationships, and ‘feelings of belonging’ (Blanchard, 2004, p. 55).

However, the science is mixed on how online community engagement may impact offline community participation. Some studies show that increased online engagement has detrimental effects on offline community participation (Kraut et al., 1998). Others, however, argue that online communities can: be more democratic in participation (Evans-Cowley and Hollander, 2010); substitute for a lack of offline community support and belonging (Lucero, 2017); and those who are active online are more likely to be engaged with their communities offline too (Stern and Dillman, 2006; Wellman et al., 2001). Thus, it is unclear how engagement with online sleep music communities may shape insomnia, influencing offline community engagement.

This is where understanding how and why people engage with online communities becomes salient. Summarizing the extensive body of research on the topic of how people engage, Malinen (2015) observes that both active participants (those who comment) and passive participants (those who browse the comments) benefit from—while also benefiting—their online communities. Algorithmic serendipity plays a crucial mediating role in this engagement by determining which content and communities people discover in the first place. When insomniacs search for sleep music or related content, YouTube’s recommendation algorithm does not simply show random results; it curates what appears based on viewing patterns, click-through rates, and engagement metrics from similar users. This means that sleepless individuals are more likely to encounter videos and comment communities where others share their experiences, creating the seemingly chance discovery of highly relevant content and like-minded communities. Both active commenters and passive browsers contribute to this process by generating the traffic and engagement data that trains the algorithm to make these targeted recommendations to other users.

Focusing on the ‘why’ of online community engagement, researchers note that some engage for entertainment (Nov et al., 2013; Nov, 2007; Blanchard and Markus, 2004), others because their social interactions with friends and family rely on the use of online platforms (Hampton, 2017), while others may be motivated to engage with online communities for self-help reasons (Salib et al., 2020). Although the reasons for online community engagement may seem deeply personal, these decisions are concurrently shaped by algorithmic serendipity, which influences not only what content people find but also how they understand what constitutes relevant or helpful material. Researchers have shown that YouTube’s algorithm reinforces existing patterns as users navigate the platform with their ‘own understanding of YouTube’s algorithmic processes’ which are then ‘embodied within their own practices, influencing modes of self-presentation, tone of voice, choice of content covered, words and sentence structures used’ (Bishop, 2018, p. 69).

## Data and methods

To create our dataset, we identified high-traffic YouTube videos—as determined by views and ‘like’ counts—focused on providing insomnia relief via relaxing music. We selected YouTube over other platforms for several methodological reasons. First, YouTube’s recommendation algorithm is particularly sophisticated in creating what we term ‘algorithmic serendipity,’ where users discover content through automated suggestions rather than intentional searches (Zhang et al., 2012; André et al., 2009; Beale, 2007). Second, the platform’s relative anonymity compared to Facebook or other similar social media sites encourages more candid self-disclosure about personal struggles like insomnia. Third, YouTube has become a dominant platform for sleep-aid content, with millions of users turning to it for insomnia relief, making it an ideal site for studying the intersection of sleeplessness and digital community formation (Eke et al., 2020). Finally, unlike platforms focused primarily on social networking, YouTube’s comment sections function more as public support communities around shared experiences, providing rich data for understanding how people understand and navigate insomnia.

We collected all the comments from the top 30 high-traffic videos using YouTube Data Tools (Rieder, 2015). This permitted us to save 420,465 comments from the YouTube Data API (Anon, 2024a), unnest them, and record both the comments and their associated ‘likes’, ‘replies’, and timestamp variables. In other words, our dataset represents a snapshot of the thirty most popular YouTube insomnia-relief music videos as of February 2024. Following the lead of Miller (2021) our video selection was both purposive and random. We searched for ‘Sleep Music’ on YouTube, then purposively sorted the recommended channels by popularity. Next, we used a random number generator to select videos for coding (Guest et al., 2006).

We conducted a systematic analysis of this data by integrating grounded theory and machine learning in a staged loop that balanced interpretive depth with scale. Informed by the inductive coding approach of Charmaz (2006) and the grounded theory methods of Strauss and Corbin (1998), our analysis proceeded through three interconnected phases that combined human interpretation with computational assistance.

We began by jointly open-coding a seed set of 500 comments, sketching provisional categories and memoing links among them. Following grounded theory principles, each member of our team brought their unique interests to the analysis, generating codes and memos that we exchanged and discussed weekly over four months to construct a common coding scheme. Simultaneously, we ran Latent Dirichlet Allocation (LDA) on the entire 420,465-comment corpus, using the algorithm’s probabilistic clusters as a surrogate “first pass” over the remaining text (Blei et al., 2003). Each topic’s top-weighted comments and keywords were exported to Excel, where we renamed clusters in sociological terms, built word-frequency tables to spot outliers, and resolved disagreements through constant comparison. This dual approach allowed us to ground our computational analysis in interpretive depth while scaling beyond what manual coding alone could achieve.

In our axial coding phase, we examined how preliminary themes interconnected, establishing logical connections between categories while continuing our human-machine analytical loop. Mis-fit comments from our LDA analysis were fed back into the model for retuning, and the revised topic labels guided a focused-coding sweep of an additional 1,500 comments. We continued in approximately 300-comment increments, alternating between LDA refinements and close reading, documenting how codes and themes connected to existing literature and detailing relationships between categories. For example, our ‘community’ theme emerged from repeated codes involving community participation, belonging, and community formation that appeared both in our manual coding and as coherent LDA topics. We maintained constant comparison throughout, identifying when this method no longer provided new insights.

In the third phase of our analyses, we focused on theoretical integration. Theoretical saturation was reached after 3,100 manually coded comments across two successive batches that yielded no new concepts. In our selective coding phase, we structured our themes into a comprehensive theory of sleeplessness, forming the primary sections of this article that connect algorithmic serendipity and the insomnia experience, disclosure practices, and community dynamics. The final, human-vetted codebook was then applied via the trained LDA model to the full dataset, giving us theme assignments for more than 100,000 comments while preserving the conceptual integrity of grounded theory.

While this approach provides access to naturalistic expressions of insomnia experiences at unprecedented scale, it carries important limitations inherent to observational and self-reported online data. Comments represent voluntary self-disclosures, made by self-selected commenters that may not reflect the full spectrum of insomnia experiences. The anonymous nature of YouTube comments, while facilitating candid disclosure, prevents verification of reported experiences or follow-up clarification. Additionally, our data captures only those who have discovered sleep music channels, excluding individuals who manage insomnia through other digital or offline means. The temporal nature of comments also means we observe single moments of expression rather than longitudinal nightly sleep patterns, limiting our ability to establish causality between community engagement and sleep outcomes.

By pairing grounded theory’s close, interpretive reading with LDA’s large-scale topic modeling, we retain qualitative depth while expanding our analysis to cover roughly 10 times more text than manual coding could reach. Throughout, we followed ethical best practices for public-comment research by stripping usernames, lightly altering titles, and avoiding verbatim quotations in our write-up (Reilly, 2013). Descriptive statistics for the corpus appear in Table 1.

**Table 1:** Sample descriptives.

	Video	Comments	Likes
1	Deep sleep	133,658	1,023,131
2	Relaxing flying sleep	133,645	1,022,945
3	Rain for sleep	28,346	188,122
4	Rain and native flutes for sleep	20,054	101,984
5	Healing sleep	18,535	280,545
6	Tibetan healing sleep	14,693	144,410
7	Release sleep music	10,488	68,919
8	Deep sleep for third eye	10,031	99,268
9	Quiet night deep sleep	8,070	71,811
10	Blue forest sleep music	7,694	130,414
11	Balancing and healing sleep	7,226	87,631
12	Calming sleep meditation	5,953	14,033
13	Black screen sleep	5,623	48,927
14	Full night sleep	2,358	39,741
15	Sleep to balance	1915	15,737
16	Fall into sleep instantly	1895	12,775
17	Sleep with inner peace	1,536	11,992
18	Theta to delta sleep	1,251	13,106
19	Sleepy falls	1,037	8,072
20	Sleep heals	999	3,792
21	Meditative sleep	906	4,082
22	Sleep for calm inner peace	828	9,160
23	Stress relief sleep	820	1903
24	Peaceful sleep	761	1,385
25	Fall asleep in 3 min	633	4,887
26	Regenerate sleep	499	2,512
27	Dolphins dreaming	285	1,044
28	Enter REM sleep	283	1,868
29	Instant sleep	248	817
30	Back to sleep	195	540

## Results

Our analysis shows that algorithmic serendipity shapes how individuals struggling to sleep experience their communities. Individual and community needs influenced associated norms of anonymous online information disclosure. As the sleep-deprived visitors to YouTube’s sleep music channels found themselves both reading about and disclosing their own experiences with the disorders present in their lives, this often prevented them from falling asleep. Instead of sleeping, they were engaging in community participation and the co-construction of belonging. In other words, paradoxically, their successful efforts to ameliorate the original factors underpinning their insomnia likely reinforce their insomnia.

### Insomnia as a trigger

Instead of merely listening to music, visitors to YouTube’s sleep music-themed channels took advantage of the anonymity provided by the platform to share their struggles with their inability to sleep. Their narratives about insomnia focused on declarations of the condition. Comments such as ‘Been an insomniac for over 2 decades, I sometimes go days without sleep desperation has brought me here,’ ‘I did not sleep at all tonight,’ and ‘I’ve really been trying to sleep, but now it’s almost 8 a.m. and I’ve been awake all night long, I’m desperate’ reflect expressions of deep need and vulnerability.

The above candid disclosures of firsthand experiences with insomnia were accompanied by declarations of intense yearning for sleep. Statements such as ‘I suffer from insomnia. All I would like is a peaceful night’ and ‘I have insomnia. I stay awake until like 5:00 a.m. G’night everyone, let us all try to get some sleep’ reflect narratives of hope intertwined with lack. These expressions of vulnerability were reciprocated with comments recounting shared experiences with insomnia, understanding, support, and well-wishes for falling asleep from the strangers reading these disclosures.

### Algorithmic serendipity

Explicit in some narratives about insomnia was the conscious awareness of the role of serendipity in finding sleep-themed music, just when the individual needed it most. Users experienced algorithmic serendipity—encountering unexpected but insomnia-relevant content through the programming of YouTube’s algorithm—in two distinct forms: *Sacred Serendipity* and *Profane Serendipity*. Sacred Serendipity is characterized by moments of profound, almost spiritual connection between individuals struggling to sleep and the content they stumbled upon. Users attributed their discovery to fate or divine intervention, as evidenced by comments like ‘03:16 a.m. in my country, I choose a random vid for sleeping and the very first I see is this… Funny how fate acts sometimes.’ This type of serendipity transcends mere coincidence, imbuing the experience with a sense of purpose and meaning.

Comments reflecting sacred serendipity revealed how select individuals interpreted their algorithmic encounters as divinely guided. One person expressed gratitude in spiritual terms:

“Thank you bro, I think the God of the heavens and the earth are speaking through you my brother, have a blessed life and stay a good human being.”

Another framed their discovery using religious language:

“Been having trouble sleeping, it's 1:30am and my man answers my prayers like the holy saint he is. Thanks for being the messiah in my time of need.”

The sense of divine timing appeared in another comment:

“I thought it was an accident that I came across this but after listening and reading these stories and comments I realize that I was greatly mistaken. Thank you.”

A similar sentiment about meaningful coincidence emerged from another user:

“Wow…I accidentally clicked into the comments (there really are no accidents… lol). So, to you, I love you. Thank you for your beautiful uplifting and encouraging words. Have a blessed day & sleep.”

These responses illustrate that when experiencing sacred serendipity, individuals feel as though the content, whether a video or comment, was meant for them in that moment, providing solace or inspiration precisely when needed. Experiences of Sacred Serendipity thus foster a deep, emotional connection with other users of the channel.

Conversely, Profane Serendipity involves the unexpected discovery of content through the mechanics of the algorithm without the layer of spiritual or significant interpretation. Users acknowledge the role of an algorithm in presenting content at opportune moments. Some expressed amusement at the timing:

“You know it's time to go to sleep when this is in your recommendation lmao.”

Others questioned the algorithm’s uncanny accuracy:

“Now someone explain to me why this is in my recommendation 5 mins after waking up from not being able to get to sleep?”

These remarks illustrate the amusement users stumbling into a sleep music-focused channel experience when consciously encountering YouTube’s algorithm.

This form of serendipity is grounded in the functionality and unpredictability of the digital platform, evoking a sense of surprise. This arises as individuals become consciously aware of YouTube’s algorithm feeding them content, and acknowledge the algorithm’s ability to offer relevant content at just the right time. One commenter noted the simultaneously unsettling and impressive nature of algorithmic timing:

“The algorithm shows it before I go to sleep, it's both scary and cool.”

Another celebrated a rare-for-them positive algorithmic experience:

“For the first time ever YouTube recommended useful content to me, best recommendation!”

Platform inconsistencies also drew commentary:

“YouTube pausing a sleep video to ask are you still watching? Idiots.”

Others observed and appreciated the broader algorithmic ecosystem:

“What a beautiful comment section… what a lovely algorithm.”

These responses illustrate that while Profane Serendipity is less imbued with personal significance compared to Sacred Serendipity, it still plays a crucial role in the modern experience of insomnia. Algorithmic serendipity offers unexpected discoveries that can lead to new insights, laughter, or simply a welcome distraction from sleeplessness.

### Community

Visitors to these channels frequently discussed that the most unexpected consequence of algorithmic serendipity for them was finding a community they deeply yearned for. Comments like ‘I came to sleep but found the most wholesome community here’ capture this discovery. Others expressed more profound connections:

“These comments give me a sense that I'm in a community, that I belong. The compassion found in the words of a stranger can revive the spirits like nothing else, I think because at its core it is a pure form of selflessness and sharing.”

These responses underscore the unexpected joy of stumbling upon a space where empathy and support flourish, facilitated by the programming of an algorithm.

Repeatedly, individuals expressed sentiments of safety and acceptance after reading the comment sections of these videos. One commenter wrote:

“If anyone ever wants to talk just message me … with this community I feel safe, I'm not anxious at all… Have a great night everyone.”

Another shared their hospital experience:

“I want to thank you all for the words of encouragement in this comment section, it's rare to see this type of community anymore. I've been in the hospital for the last few weeks, and some days it's hard to find peace with all the negatives… we are here together … I call this thread my family.”

A third described finding solace in moments of profound loneliness:

“I was quite literally at the loneliest I have ever been. Then I saw what you all wrote. I can feel your love, sincerity. Even if it is for a moment, I know that I am not alone, I am not gasping for breath, I feel peace. I love you all.”

Instead of finding sanctuary via the unconsciousness of biological sleep, over and over, insomniacs disclosed that they were awake as they were looking for sanctuary in a family, in a community.

This sense of belonging and mutual support then became a lifeline, especially for those going through challenging times. Anonymous users would ‘check-in’ with each other via the comment sections in these videos, despite their inability to directly ‘tag’ an individual about whom they were concerned. In many instances, an original poster would respond months or years later, overwhelmed by the unexpected expressions of support from strangers. This would be followed by concerned users replying, after monitoring the comments while patiently waiting for a reply for an extended period of time.

### Community sustenance

The results of our LDA topic modeling show that these sleep communities are sustained through five broad topics and two distinct roles played by community members. Each topic encompasses a range of terms that resonate with community members. The first topic, ‘Gratitude,’ is characterized by expressions of thanks and blessings, often symbolized through the folded hands (prayer) emoji. This topic reveals a common sentiment of appreciation within the community—whether for the sleep or life-related advice received or the supportive concern of others. Such expressions of gratitude often elicited reciprocal expressions of well-wishes. This fostered a positive atmosphere in the comments accompanying these videos, reinforcing community bonds and encouraging continued engagement.

The second topic, ‘Love’ is characterized by expressions of affection (strangers telling each other ‘I love you’ or ‘I love you all’), kindness, noting that everyone in the comment section is ‘so cool,’ along with the heart emoji. This suggests an environment where people feel a strong emotional connection to each other, promoting an inclusive and caring online community where anonymous individuals can express love and receive the same in return.

In the third topic, ‘Therapeutic Effects,’ terms like ‘help,’ ‘calming,’ ‘glad,’ ‘sleep,’ and ‘kind’ emerge, highlighting the therapeutic nature of sleep music-based online community interactions. Discussions and content that provide help or calming advice can have therapeutic effects on individuals struggling with sleep issues, offering a form of support that goes beyond mere conversation, providing physical and emotional reprieve.

‘Sound Effects’ form the fourth topic, with words such as ‘beautiful’ and ‘great,’ accompanied by smiling face emojis. This topic focuses on the direct experience of the music, highlighting the importance of auditory content in influencing both mood and sleep quality. These terms suggest that community members value the positive sensory impact and ambient sounds of sleep music.

Lastly, the ‘Positive Reactions’ topic encompasses responses such as ‘wow,’ the ‘smiling face with hugging hands emoji,’ ‘sparkles emoji,’ and ‘perfect,’ indicating moments of joy and surprise. These may be elicited by the discovery of unexpectedly pleasant sounds and helpful content, including a sense of amazement at the kindness of anonymous strangers. Together, these topics describe a digital ecosystem where sleepless individuals find not just ambient sounds and unsolicited sleep advice from strangers, but a supportive community where they can experience the full spectrum of human connection, from gratitude and love to therapeutic interactions. Table 2 shows these topics and associated terms.

**Table 2:** LDA topics, associated terms, corpus prevalence percentage.

	Topic	Terms	Prevalence (%)
1	Gratitude	thank; bless; folded hands emoji	25.15
2	Love	love; kind; cool; heart emoji	17.09
4	Therapeutic effects	help; calming; glad; sleep; kind	19.54
3	Sound Effects	beautiful; great; smiling face emoji	19.29
5	Positive Reactions	wow; smiling face with hands emoji; sparkes; perfect	18.93

Engaged individuals primarily assume two key roles in these communities: Supporter and Supportee. The topics Supporters generate include ‘Solidarity,’ ‘Positivity,’ ‘Manifestation,’ ‘Kindness,’ ‘Wisdom,’ and ‘Light.’ The topics generated by Supportees include ‘Rumination,’ ‘Negativity,’ ‘Sadness,’ ‘Shame,’ ‘Self-Pity,’ and ‘Self-Doubt.’ The common quality between these two roles is the anonymous sharing of deeply personal individual experiences with strangers. This encompasses both the sharing of negativity through comparisons of hardships and unpleasant experiences, and the sharing of positivity and encouragement to ‘keep going.’

The combination of our LDA topic modeling and grounded theory themes highlight how the experience of algorithmic serendipity can serve as a conduit: guiding individuals toward the co-construction of communities they are deeply lacking. In the anonymous comment sections of these sleep music videos, they find solace, understanding, and a sense of family. This demonstrates the unexpected yet profound ways in which technology can facilitate human connection, even when we should be sleeping.

### Disclosure

#### Physical disorders

An outcome of sleep-deprived individuals finding a supportive community in the comment sections of sleep music videos is that they felt secure in anonymously sharing the troubles sustaining their insomnia. Their narratives can be sorted into three distinct themes: Disclosures of Physical Disorders, Mental Disorders, and Environmental Disorders. Anonymous disclosures of physical disorders reflected a wide range of physical health challenges, from chronic and terminal illnesses and surgery complications to the physical repercussions of medical treatments along with associated fears and pains. One commenter dealing with multiple serious conditions shared:

“I have a hard time sleeping most nights because I have metastatic breast cancer, spread to my bones and hypothyroid. And my hubby is ill with stiff persons syndrome, I don't know how much time we have left … Thank you for the company.”

Another described their post-surgical experience:

“Wish me luck sleeping tonight. I had six reconstructive surgeries from breast cancer today! Liposuction. Fat grafting in both breasts, scar removals, he made a nipple (idk exactly how).”

Fear related to cardiac surgery emerged in another disclosure:

“What if I don't have a tomorrow ever? Since I had heart surgery, I fear my life will be cut short. Someday my heart pounds like it's going to fall out while I sleep. I'm scared to sleep at night.”

One person described the intersection of disability, pain, and financial stress:

“I am alone, disabled and pain filled, not able to work enough hours to support myself. Sorrow, fear, and anger. This is helping me unplug from these horrid thoughts. Perhaps I can sleep.”

While many of the physical disorders listeners disclosed were quite serious, others were easier to manage, as one commenter noted:

“I'm reading this at 22: 20, and I'm starting to get sleepy. I came here because I had a headache, it's all better now, goodnight.”

The above comments underscore that physical ailments contribute to sleep disturbances. Yet, physical ailment-based narratives extend beyond mere accounts of medical conditions; they reflect the struggles of individuals grappling with the uncertainty of life, the fear of mortality, and the isolation that often accompanies illness. The act of sharing these experiences in a public, yet anonymous, forum highlights a collective search for understanding and community among those experiencing insomnia due to physical hardships. The comments reflect a diversity of experiences but are united by a common theme: the quest for some semblance of normalcy via the luxury of simply falling asleep, finding comfort in virtual co-sleeping rituals animated by sleep music.

#### Mental disorders

In addition to disclosing how physical disorders disturbed their sleep, many sleep music video listeners also anonymously disclosed their experiences with mental disorders. Mental Disorder-based insomnia disclosures revealed how psychological struggles intersected with sleeplessness. One commenter described the cycle of anxiety and sleeplessness:

“I've developed serious social anxiety and depression… I have terrible insomnia and spend nights staring at a wall.”

Another connected grief and cognitive concerns:

“My mother passed from dementia and now I feel like I'm getting it. I have major sleep deprivation and my mind is foggy.”

Pet loss emerged as a significant trigger for sleep disruption, as one person shared:

“After a resurgence in insomnia after my dog died, I found these and have finally been able to start sleeping again. Sweet dreams, y'all and thanks for being so kind to one another.”

Multiple traumatic losses compounded sleep difficulties for another commenter:

“I went through a divorce, then my sister was diagnosed with leukemia and passed away. My friend died of brain cancer 4 months later. I moved changed jobs and have anxiety, depression, and I can't sleep.”

Long-term struggles with both mental health and economic concerns appeared in another disclosure:

“Insomniac since forever … Going through depression and fighting it each day … I wish you all a blessed life … Please pray for me that I am employed by July.”

The comments highlight that these experiences are not just about mental health disorders in isolation but about how these conditions exacerbate insomnia, creating a vicious cycle. The comment sections of these videos allowed strangers to engage in community building by sharing struggles and offering encouragement, support, and advice to each other. Sleep-deprived individuals repeatedly asserted that the virtual community they stumbled into in the comment sections of these videos offers more than just a space to vent; it provides a sense of belonging and collective empathy that many lack in their offline lives. The online companionship they found underscores the human need for connection and understanding. Through sharing their Mental Disorder-based insomnia, these individuals actively work on co-creating a unique community, highlighting the transformative power of anonymously disclosing deeply personal experiences online.

#### Environmental disorders

Anonymous disclosures of environmental disorders reflected a wide range of space-based challenges, from living in war zones, noisy neighbors, family member and animal sounds, to traffic noises preventing individuals from sleeping. One person in an active conflict zone wrote:

“Thanks for this, it really helps me sleep because my city was just bombed.”

Insect noise presented another environmental challenge:

“Mosquito noise here all night, this music saved my life.”

Public transportation created sleeping difficulties for someone without stable housing:

“Trying to drown out the noise on public transit @ 4am so I can catch two more hours of sleep.”

Neighbor-generated noise emerged as a persistent problem for sensitive sleepers:

“I moved to a new apartment and my neighbors are loud … had months without sufficient sleep and had many crying episodes because I'm a light sleeper and a highly sensitive person… because of this I can sleep better.”

One commenter described experiencing heightened auditory sensitivity:

“I use this music to block out a frequency that isn't audible to everyone and can't be recorded by any sound recorder because it's actually a form of infrasound which is only usually heard by animals and young children. I find it torturous to the point of complete despair especially at night when there is no outside noise to diffuse the sound.”

The comments relating to Environmental Disorder-based insomnia expose the multifaceted relationship between societal structures, personal agency, and sleep practices. Individuals use sleep music to construct more peaceful sleep environments amidst various environmental disturbances. The disclosures about struggling to sleep due to bombs, animal noises, traffic sounds, loud neighbors, and high-pitched frequencies underscore that the social experience of insomnia transcends individual biological or psychological needs. These environmental factors function as external stressors that disrupt sleep, reflecting broader issues around urbanization, conflict, and technological intrusion into personal spaces. Relying on sleep music videos as a coping mechanism shows how individuals exercise agency within these constraints. By looking for and sharing encouragement and advice in online communities, they not only seek to mitigate the impact of these disturbances on their sleep but also create a shared space for collective resilience. This dynamic interaction between structure and agency in the context of insomnia underscores the importance of viewing sleep as a sociological phenomenon while considering the socio-environmental factors influencing sleep practices.

### Insomnia as a consequence

Thus far, our results revealed that the unexpected encounters facilitated by algorithms play a crucial role in shaping the experiences of individuals seeking solace from insomnia via sleep music on YouTube. We illustrated that the personal and collective needs of sleep-deprived individuals not only shape the norms around anonymously sharing extremely private information online but also encourage them to engage with each other. As visitors to YouTube’s sleep music channels empathize with others’ insomnia narratives and share their own struggles, they participate in community building. This activity, rooted in the search for understanding and connection, paradoxically perpetuates the very sleeplessness they seek to escape.

Comments showing the full-circle of insomnia illustrate the above paradox. One person acknowledged choosing community over sleep:

“It's 6:17am here. I haven't slept because the comment section here is more welcoming than home. lmao”

Another tried to redirect fellow insomniacs back to their original purpose for listening to relaxing music:

“Whoever is reading this… Dude! try to sleep instead of reading these comments!”

Some noted that the comments provided essential emotional support:

“Didn't know how much I needed to read these comments. Thanks everyone… I needed these comments to remind me to remember who I am. I am born to be love and light in dark places. Getting myself back”

Others described how the openness of strangers created a dream-like state:

“The comments are so warm and kind. Reading all of the comments kinda puts me in a dream state because I never knew that people could really be this open to share their life and experiences. I know I'm not alone. Thank you everyone”

The comfort found in shared struggle appeared in another response:

“Had another sleepless night. I just want to sleep in my mother's lap worry free. Reading all your comments made me feel positive, there are so many folks fighting every day, still sending out prayers and wishes… God bless my bros and sis.”

These responses demonstrate the central paradox: the online platforms intended to facilitate sleep become spaces that sustain wakefulness through community connection.

**Figure 1:**
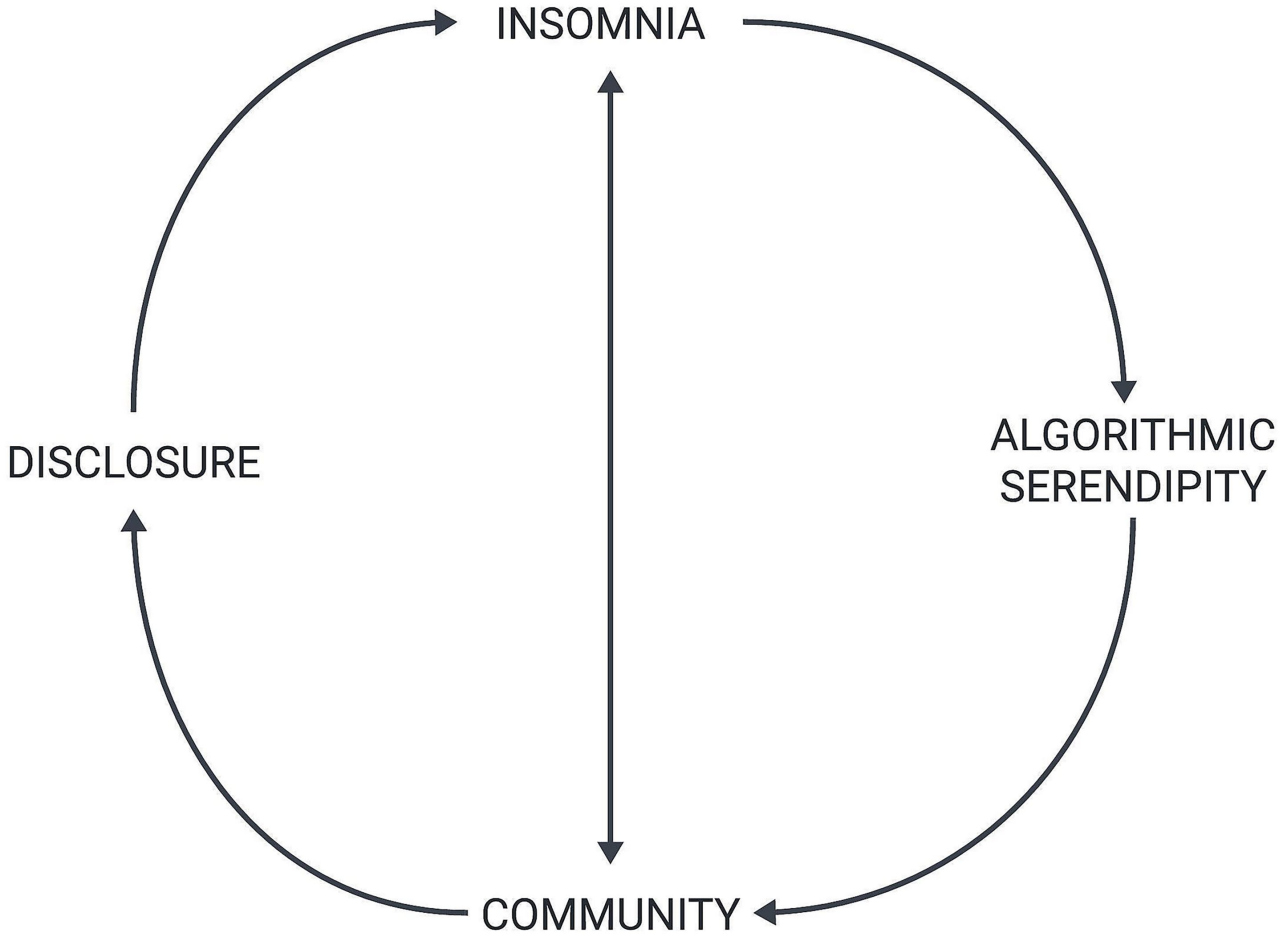
The paradox of insomnia in the digital age.

Figure 1 summarizes the sociological paradox of our collective insomnia in the modern age. As the figure shows, insomnia creates the conditions for the algorithmic serendipity experienced by sleep music video users. Algorithmic serendipity then shapes how individuals struggling to sleep find and co-create online communities. Their individual and community-based needs influence associated norms of online information disclosure. As these sleep-deprived music listeners unexpectedly find themselves both reading about and disclosing their own experiences with the various disorders causing their insomnia, this prevents them from falling asleep. Lacking supportive offline communities, instead of sleeping, they engage in community participation and the co-construction of belonging. Counterintuitively, their successful efforts to ameliorate the original factors undergirding their insomnia perpetuate their insomnia.

## Discussion

This study examined the sociological conundrum of sleeplessness. Integrating literature from the biopsychology and sociology of sleep, research from science and technology studies and online community participation, while drawing on over 400,000 comments from high-traffic YouTube videos, we demonstrate that algorithmic serendipity shapes how individuals struggling to sleep understand and interact with their communities. As insomniacs experience feelings of isolation from their offline support networks due to their condition (Kyle et al., 2010), the line between their offline and online communities blurs (Wilson and Peterson, 2002): online communities are central for how individuals struggling with sleeplessness find belonging and construct identities by actively enacting two distinct community roles. Although many individuals were aware of algorithms guiding them to sleep-themed music channels online, they frequently noted that an unexpected consequence of algorithmic serendipity was finding a community they desperately needed. This new-found belonging and reciprocal support became a vital source of comfort for insomnia sufferers. A crucial outcome of sleep-deprived individuals finding a supportive community in the comment sections of sleep music videos is that they felt secure in anonymously sharing the disorders sustaining their insomnia.

Blurring the distinctions between public-private and individual-collective spheres (Crary, 2013; Williams, 2011), individuals remained awake to share harrowing experiences with community disorganization, physical health challenges, and struggles with mental disorders. For many community members, this was their first experience of recognition as strangers extended support and shared similar experiences, fostering a sense of belonging. The personal and collective needs of sleep-deprived individuals guided disclosure norms while also encouraging continued community engagement.

However, the relationship between online community engagement and sleeplessness reveals a complex tension. While some explicitly acknowledged choosing community connection over sleep, others found that the sense of belonging and emotional support helped them feel more settled and centered—qualities that could potentially counteract insomnia (Kim et al., 2021; Robbins et al., 2019; Lucero, 2017). This creates a paradox, where the very online communities that provide emotional relief from the isolation of insomnia may also sustain wakefulness through their engaging nature. Rather than simply exacerbating sleeplessness, these online spaces represent a trade-off: individuals may sacrifice immediate sleep for longer-term emotional well-being and community connection. Online insomnia-oriented communities that ease loneliness by fostering belonging also keep participants awake, complicating any straightforward assessment of their net effect on sleep.

Our findings are noteworthy, as the interactional blueprints and community dynamics sustaining online co-sleeping communities can have unexpected outcomes in other domains. For instance, while many insomniacs struggle with day-to-day social participation (Kyle et al., 2010), during the night, they are educating themselves about their condition while concurrently engaging in active community building. This happens when they are in a vulnerable state, characterized by both sleep deprivation and emotional arousal, bolstering the disclosure of deeply personal experiences and medical conditions (Fernandez-Mendoza and Vgontzas, 2013). The lifestyle and medical advice received from strangers during the night may conflict from the medical advice their providers impart. Further, as these online communities are global, many insomniacs become aware of the medical and workplace resources provided to individuals living in differing contexts from their own. This could influence their offline institutional expectations (Vedaa et al., 2016).

Our findings delineate an agenda the next round of scholars must confront. Since the sample we rely on is anonymous, we could not control for individual sociodemographic characteristics such as age. Future research should examine how the experience of sleeplessness differs across the life course. This is especially important to consider in younger ages, as virtual co-sleeping communities may allow for the construction of new identities during the formidable life course moment of adolescence (Wilson and Peterson, 2002). More work is needed on both the social causes and consequences of sleeplessness, focusing on how they differ in dissimilar contexts and the ways in which they are shaped by gender, race, and income. Further, it is vital that future studies consider how the navigation and mitigation strategies of insomniacs are undergirded by their cultural contexts, remaining mindful of both online and offline social network influences.

Although we draw from a global platform, our sample is not representative of the general population. Thus, it would be prolific to supplement this work with robust, nationally representative surveys along with interviews exploring the social experience of insomnia. Additionally, future research should consider alternative explanations for the patterns observed in our data. For instance, people with insomnia may seek out online communities on their own. Without representative survey data, it is hard to quantify the exact influences of personal need versus algorithmic suggestion. Furthermore, the way people express themselves in the comments may be shaped by YouTube’s norms, rather than directly reflecting their general modes of expression and insomnia experiences. These considerations highlight the need for more nuanced investigations into the relationship between platform dynamics and user behavior.

Knowing that insomnia is detrimental for community participation (Kim et al., 2021; Robbins et al., 2019) and that online community engagement shapes offline community participation (Kraut et al., 1998; Wellman et al., 2001; Stern and Dillman, 2006; Evans-Cowley and Hollander, 2010), sleep researchers also need to grapple with the interaction of the two. We could expect that the social experience of insomnia would have ripple effects across social networks. Thus, future work should also examine how sleeplessness shapes social network composition and size. It would also be enlightening to consider the social contagion of insomnia, keeping algorithmic serendipity in mind, while remaining open to alternative mechanisms that may better explain the observed phenomena.

Our results underscore that the consideration of algorithmic serendipity and community engagement is helpful for understanding the sociological paradox of insomnia in the digital age. To our knowledge, prior studies have not examined the relationship between sleeplessness, agency as it is shaped by algorithmic serendipity, and community engagement. Our work is an important first step in uncovering how engagement with online sleep music communities may shape insomnia. We show that algorithmic serendipity influences how individuals struggling to sleep discover and create online co-sleeping communities, with unforeseen consequences for their condition. Our work underscores the need for comprehensive, population level studies on how digital technology shapes sociotemporal experiences undergirding individual and community well-being outcomes. Research on algorithmic serendipity and the sociology of sleeplessness carries substantial health and social policy implications.

## Data Availability

Publicly available datasets were analyzed in this study. This data can be found at: www.BorokaBo.com.

## References

[ref1] André P. Schraefel M. C. Teevan J. Dumais S. T. (2009). Discovery is never by chance: designing for (un)serendipity. Proceedings of the seventh ACM conference on creativity and cognition 305–314.

[ref2] Anon (2024a) YouTube data API: YouTube API developers Guide Available at: https://developers.google.com/youtube/2.0/developers_guide_protocol_audience (Accessed May 2, 2024).

[ref3] Anon (2024b) National Sleep Foundation. Available at: https://www.sleepfoundation.org (Accessed April 22, 2024).

[ref4] Araújo T. Jarrin D. C. Leanza Y. Vallières A. Morin C. M. (2017). Qualitative studies of insomnia: current state of knowledge in the field. Sleep Med. Rev. 31, 58–69. doi: 10.1016/j.smrv.2016.01.003, PMID: 27090821 PMC4945477

[ref5] Beale R. (2007). Supporting serendipity: using ambient intelligence to augment user exploration for data mining and web browsing. Int. J. Hum.-Comput. Stud. 65, 421–433. doi: 10.1016/j.ijhcs.2006.11.012

[ref6] Bishop S. (2018). Anxiety, panic and self-optimization: inequalities and the YouTube algorithm. Convergence 24, 69–84. doi: 10.1177/1354856517736978

[ref7] Blanchard A. (2004). The effects of dispersed virtual communities on face-to-face social capital. In Social capital and information technology. Eds., M. Huysman and V. Wulf, Cambridge: MIT Press.

[ref8] Blanchard A. L. Markus M. L. (2004). The experienced sense of a virtual community: characteristics and processes. ACM Sigmis Datab. 35, 64–79. doi: 10.1145/968464.968470

[ref9] Blei D. M. Ng A. Y. Jordan M. I. (2003). Latent dirichlet allocation. J. Mach. Learn. Res. 3, 993–1022. doi: 10.5555/944919.944937

[ref10] Buysse D. J. Ancoli-Israel S. Edinger J. D. Lichstein K. L. Morin C. M. (2006). Recommendations for a standard research assessment of insomnia. Sleep 29, 1155–1173. doi: 10.1093/sleep/29.9.1155, PMID: 17040003

[ref11] Charmaz K. (2006). Constructing grounded theory: a practical guide through qualitative analysis. Thousand Oaks, CA: Sage.

[ref12] Chilcott L. A. Shapiro C. M. (1996). The socioeconomic impact of insomnia: an overview. PharmacoEconomics 10, 1–14. 10.2165/00019053-199600101-00003 10163422

[ref13] Cordi M. J. Ackermann S. Rasch B. (2019). Effects of relaxing music on healthy sleep. Sci. Rep. 9:9079. doi: 10.1038/s41598-019-45608-y, PMID: 31235748 PMC6591240

[ref14] Coveney C. Greaney M. Hsu E. L. Meadows R. Williams S. J. (2023). Technosleep: Frontiers, fictions, futures. Cham, Switzerland: Springer Nature.

[ref15] Crary J. (2013). 24/7: Late capitalism and the ends of sleep. London, UK: Verso Books.

[ref16] Daley M. Morin C. M. LeBlanc M. Grégoire J.-P. Savard J. (2009). The economic burden of insomnia: direct and indirect costs for individuals with insomnia syndrome, insomnia symptoms, and good sleepers. Sleep 32, 55–64., PMID: 19189779 PMC2625324

[ref17] Dickson G. T. Schubert E. (2019). How does music aid sleep? Literature review. Sleep Med. 63, 142–150. doi: 10.1016/j.sleep.2019.05.016, PMID: 31655374

[ref18] Eke R. Li T. Bond K. Ho A. Graves L. (2020). Viewing trends and users’ perceptions of the effect of sleep-aiding music on YouTube: quantification and thematic content analysis. J. Med. Internet Res. 22:e15697. doi: 10.2196/15697, PMID: 32831182 PMC7477671

[ref19] Espie C. A. (2022). The ‘5 principles’ of good sleep health. J. Sleep Res. 31:e13502. doi: 10.1111/jsr.13502, PMID: 34676592 PMC9285041

[ref20] Evans-Cowley J. Hollander J. (2010). The new generation of public participation: internet-based participation tools. Plann. Pract. Res. 25, 397–408. doi: 10.1080/02697459.2010.503432

[ref21] Fernandez-Mendoza J. Vgontzas A. N. (2013). Insomnia and its impact on physical and mental health. Curr. Psychiatry Rep. 15, 1–8. doi: 10.1007/s11920-013-0418-8 PMC3972485 24189774

[ref22] Giddens A. (2020). “Modernity and self-identity: self and society in the late modern age” in The new social theory reader. Eds., S. Seidman and J. C. Alexander (London UK: Routledge), 354–361.

[ref23] Guest G. Bunce A. Johnson L. (2006). How many interviews are enough? An experiment with data saturation and variability. Field Methods 18, 59–82. doi: 10.1177/1525822X05279903

[ref24] Hampton K. N. (2017). Studying the digital: directions and challenges for digital methods. Annu. Rev. Sociol. 43, 167–188. doi: 10.1146/annurev-soc-060116-053505

[ref25] Harvey A. G. (2001). Insomnia: symptom or diagnosis? Clin. Psychol. Rev. 21, 1037–1059. doi: 10.1016/S0272-7358(00)00083-0 11584515

[ref26] Henry D. McClellen D. Rosenthal L. Dedrick D. Gosdin M. (2008). Is sleep really for sissies? Understanding the role of work in insomnia in the US. Soc. Sci. Med. 66, 715–726. doi: 10.1016/j.socscimed.2007.10.007, PMID: 18006129

[ref27] Hillman D. R. Lack L. C. (2013). Public health implications of sleep loss: the community burden. Med. J. Aust. 199, S7–S10. doi: 10.5694/mja13.10620, PMID: 24138358

[ref28] Kim D. E. Roberts T. J. Moon C. (2021). Relationships among types of activity engagement and insomnia symptoms among older adults. BMC Geriatr. 21:87. doi: 10.1186/s12877-021-02042-y 33516192 PMC7847011

[ref29] Koh J. Kim Y.-G. Butler B. Bock G.-W. (2007). Encouraging participation in virtual communities. Commun. ACM 50, 68–73. doi: 10.1145/1216016.1216023

[ref30] Kraut R. Patterson M. Lundmark V. Kiesler S. Mukophadhyay T. Scherlis W. (1998). Internet paradox: a social technology that reduces social involvement and psychological well-being? Am. Psychol. 53, 1017–1031. doi: 10.1037/0003-066X.53.9.1017, PMID: 9841579

[ref31] Kyle S. D. Espie C. A. Morgan K. (2010). ‘…not just a minor thing, it is something major, which stops you from functioning daily’: quality of life and daytime functioning in insomnia. Behav. Sleep Med. 8, 123–140. doi: 10.1080/15402002.2010.487450 20582756

[ref33] Léger D. Massuel M.-A. Metlaine A. SISYPHE Study Group (2006). Professional correlates of insomnia. Sleep 29, 171–178., PMID: 16494084

[ref1002] Léger D. Bayon V. (2010). Societal costs of insomnia. Sleep medicine reviews, 14, 379–389. doi: 10.1016/j.smrv.2010.01.003, PMID: 20359916

[ref34] Levinas E. (1978). Existence and existents. The Hague: Martinus Nijhoff.

[ref35] Lieblich A. Zilber T. B. Tuval-Mashiach R. (2008). Narrating human actions: the subjective experience of agency, structure, communion, and serendipity. Qual. Inq. 14, 613–631. doi: 10.1177/1077800408314352

[ref36] Lucero L. (2017). Safe spaces in online places: social media and LGBTQ youth. Multicult. Educ. Rev. 9, 117–128. doi: 10.1080/2005615X.2017.1313482

[ref37] Malinen S. (2015). Understanding user participation in online communities: a systematic literature review of empirical studies. Comput. Human Behav. 46, 228–238. doi: 10.1016/j.chb.2015.01.004

[ref38] Matin K. (2021). “Deciphering the modern Janus: societal multiplicity and nation-formation” in Multiplicity (London, UK: Routledge), 40–55.

[ref39] Meadows C. (2019). “One of these days I'M going to get Organiz-Ized” in Insomnia as the arrhythmic experience of modernity. Toronto, CA: York University.

[ref40] Miller V. (2020) "Understanding Digital Culture. Kent, UK: Sage Publications.

[ref41] Miller D. T. (2021). Characterizing qanon: analysis of YouTube comments presents new conclusions about a popular conservative conspiracy. First Monday. doi: 10.5210/fm.v26i2.10168

[ref43] Nakata A. Haratani T. Takahashi M. Kawakami N. Arito H. Kobayashi F.. (2004). Job stress, social support, and prevalence of insomnia in a population of Japanese daytime workers. Soc. Sci. Med. 59, 1719–1730. doi: 10.1016/j.socscimed.2004.02.002, PMID: 15279928

[ref44] Nov O. (2007). What motivates Wikipedians? Commun. ACM 50, 60–64. doi: 10.1145/1297797.1297798

[ref45] Nov O. Arazy O. López C. Brusilovsky P. (2013) "Exploring personality-targeted UI Design in Online Social Participation Systems." Proceedings of the SIGCHI conference on human factors in computing systems. 361–370.

[ref46] Ohayon M. M. (2002). Epidemiology of insomnia: what we know and what we still need to learn. Sleep Med. Rev. 6, 97–111. doi: 10.1053/smrv.2002.0186, PMID: 12531146

[ref47] Ong K.-L. Leao S. Krezel A. (2014). Participatory sensing and education: helping the community mitigate sleep disturbance from traffic noise. Int. J. Pervasive Comput. Commun. 10, 419–441. doi: 10.1108/IJPCC-04-2014-0030

[ref48] Putnam R. D. (1995). Tuning in, tuning out: the strange disappearance of social capital in America. PS Polit. Sci. Polit. 28, 664–683. doi: 10.2307/420517

[ref49] Reilly P. (2013). The ‘Battle of stokes croft’ on YouTube: The development of an ethical stance for the study of online comments. Cham: Sage.

[ref50] Rieder B. (2015) YouTube data tools. Available at: https://github.com/bernorieder/YouTube-Data-Tools (Accessed April 1, 2024).

[ref51] Robbins R. Jean-Louis G. Gallagher R. A. Hale L. Branas C. C. Gooneratne N.. (2019). Examining social Capital in Relation to sleep duration, insomnia, and daytime sleepiness. Sleep Med. 60, 165–172. doi: 10.1016/j.sleep.2019.03.019, PMID: 31175050 PMC6642663

[ref52] Rosekind M. R. Gregory K. B. (2010). Insomnia risks and costs: health, safety, and quality of life. Am. J. Manag. Care 16, 617–626. 20712395

[ref53] Roth T. Roehrs T. (2003). Insomnia: epidemiology, characteristics, and consequences. Clin. Cornerstone 5, 5–15. doi: 10.1016/s1098-3597(03)90031-7, PMID: 14626537

[ref54] Rotman D. Golbeck J. Preece J. (2009). "The community is where the rapport is--on sense and structure in the Youtube community." Proceedings of the fourth international conference on Communities and technologies: 41–50.

[ref55] Salib G. Gorichanaz T. Agosto D. E. (2020). “Dear a my”: seeking support on YouTube. Proc. Assoc. Inf. Sci. Technol. 57:e273. doi: 10.1002/pra2.273

[ref1001] Schor J. (2008). The overworked American: The unexpected decline of leisure. New York, NY: Basic Books.

[ref56] Soldatos C. R. Allaert F. A. Ohta T. Dikeos D. G. (2005). How do individuals sleep around the world? Results from a single-day survey in ten countries. Sleep Med. 6, 5–13. doi: 10.1016/j.sleep.2004.10.006, PMID: 15680289

[ref57] Stepanski E. J. Wyatt J. K. (2003). Use of sleep hygiene in the treatment of insomnia. Sleep Med. Rev. 7, 215–225. doi: 10.1053/smrv.2001.0246, PMID: 12927121

[ref58] Stern M. J. Dillman D. A. (2006). Community participation, social ties, and use of the internet. City Community 5, 409–424. doi: 10.1111/j.1540-6040.2006.00191.x

[ref59] Strauss A. Corbin J. (1998). Basics of qualitative research techniques: techniques and procedures for developing grounded theory. Newbury Park, CA: Sage.

[ref60] Taylor B. (1993). Unconsciousness and society: the sociology of sleep. Int. J. Polit. Cult. Soc. 6, 463–471. doi: 10.1007/BF01415970

[ref61] Van Someren E. J. W. (2021). Brain mechanisms of insomnia: new perspectives on causes and consequences. Physiol. Rev. 101, 995–1046. doi: 10.1152/physrev.00046.2019 32790576

[ref62] Vedaa Ø. Krossbakken E. Grimsrud I. D. Bjorvatn B. Sivertsen B. Magerøy N.. (2016). Prospective study of predictors and consequences of insomnia: personality, lifestyle, mental health, and work-related stressors. Sleep Med. 20, 51–58. doi: 10.1016/j.sleep.2015.12.002, PMID: 27318226

[ref64] Walker M. (2017). Why we sleep: unlocking the power of sleep and dreams. New York, NY, USA: Simon and Schuster.

[ref65] Wellman B. Haase A. Q. Witte J. Hampton K. (2001). Does the internet increase, decrease, or supplement social capital? Social networks, participation, and community commitment. Am. Behav. Sci. 45, 436–455. doi: 10.1177/00027640121957286

[ref66] Williams S. J. (2002). Sleep and health: sociological reflections on the dormant society. Health 6, 173–200. doi: 10.1177/136345930200600203

[ref67] Williams S. J. (2008). The sociological significance of sleep: progress, problems and prospects. Sociol. Compass 2, 639–653. doi: 10.1111/j.1751-9020.2007.00088.x

[ref68] Williams S. (2011). The politics of sleep: Governing (un) consciousness in the late modern age. New York, NY, USA: Springer.

[ref69] Williams S. J. Boden S. (2004). Consumed with sleep? Dormant bodies in consumer culture. Sociol. Res. Online 9, 98–109. doi: 10.5153/sro.914

[ref70] Wilson S. M. Peterson L. C. (2002). The anthropology of online communities. Annu. Rev. Anthropol. 31, 449–467. doi: 10.1146/annurev.anthro.31.040402.085436

[ref71] Wolf-Meyer M. (2008). Sleep, signification and the abstract body of allopathic medicine. Body Soc. 14, 93–114. doi: 10.1177/1357034X08096897

[ref72] Zhang Y. C. Ó Séaghdha D. Quercia D. Jambor T. (2012). Auralist: introducing serendipity into music recommendation. In Fifth ACM International Conference on Web Search and Data Mining (13–22).

